# Israel is failing to protect its citizens from secondhand smoke: underestimating public support

**DOI:** 10.1186/2045-4015-2-24

**Published:** 2013-06-27

**Authors:** Stanton A Glantz

**Affiliations:** 1 Center for Tobacco Control Research and Education, University of California, San Francisco, San Francisco, CA 94143-1390, USA

## Abstract

Rather than clearly and unequivocally requiring 100% smokefree workplaces and public places (including restaurants, bars and other entertainment venues), Israeli law contains several elements that parallel the tobacco companies’ “accommodation” program, which is designed to maintain the social acceptability of smoking and protect industry profits. Rather than 100% smokefree workplaces, smoking is permitted in private offices despite the fact that it then wafts throughout the building. Bars and pubs are allowed to set aside a quarter of their space for smokers, as long as it is in a separate room, and this explains the dangerous levels of secondhand smoke air pollution in Israeli bars and pubs. The weaknesses in the current Israeli laws are sending Israeli citizens to the hospital for secondhand smoke-induced heart attacks, asthma and other diseases. The Israeli government needs to catch up with the rest of the developed world and enact and implement a strong smokefree law.

This is a commentary on http://www.ijhpr.org/content/2/1/20/.

## Commentary

The most amazing statistic Laura Rosen and colleagues report in their paper “Do health policy advisors know what the public wants? An empirical comparison of how health policy advisors assess public preferences regarding smoke-free air, and what the public actually prefers” [1] is that 27% of Israel’s *health policy advisors* were exposed to secondhand smoke *at work*. (This is better than for the public, 46% of whom suffered secondhand smoke exposure at work). The fact that even workplaces for health care advisors are not 100% smokefree reflects the fact that the government is not effectively protecting Israeli citizens from the thousands of toxic chemicals in secondhand smoke and the heart disease and cancer they cause.

An objective measure of this failure is the fact that third of Israeli nonsmokers have detectable levels of cotinine (a metabolite of nicotine) in their urine, a result that has been attributed to poor enforcement of Israel’s smoking restrictions [2].

But the problem goes much deeper than that, to the law itself. Rather than clearly and unequivocally requiring 100% smokefree workplaces and public places (including restaurants, bars and other entertainment venues), Israeli law permits smoking in private offices despite the fact that smoke then wafts throughout the building and allows bars and pubs to set aside a room with a quarter of their space for smokers.

These are exactly the policies that multinational tobacco companies, including Philip Morris [3], want written into law to keep smoking socially acceptable and maintain cigarette consumption and their profits. Faced with the fact that opposing all restrictions on smoking in restaurants and bars was no longer working, in 1989 Philip Morris recognized that it could maintain the social acceptability of smoking by supporting the creation of smoking and nonsmoking areas, which led it to being promoting its “accommodation” program [4-9] in hospitality venues. While effective at protecting tobacco company interests, the smoke still moves out of the smoking areas, which explains the dangerous levels of secondhand smoke air pollution measured in Israeli bars, pubs, and cafes despite the nominal smoking restrictions [10].

Rather than focusing on confronting the core problems with the current law, the government has pursued smokefree entrances to health facilities and train platforms. While such restrictions are a good idea because cigarettes pollute the air outdoors too [11-13], these problems wane in comparison to the widespread smoking indoors.

Israel’s weak law is particularly surprising, since the global pattern of national smokefree laws has involved a leading wave of high-income, developed countries enacting laws, with lower-income countries following [14-16]. The Israeli government seems to be following the lead of the Netherlands [17], whose Ministry of Health undermined implementation of their smokefree bar law, and poor countries with low state capacity that adopt smokefree laws then fail to enforce them [18]. Israel is even lagging behind Latin America, which has gone from zero out of 20 countries in 2003 to twelve countries in 2013 [19,20].

To stay out of the public eye when opposing smokefree policies, in many countries around the world the tobacco companies work through “smokers’ rights” groups [21,22] created by the industry, restaurant associations [4], bar associations [4,23], gambling interests [24], the alcohol industry [25], and interest groups on the right [26] and left [27-29]. Rosen et. al. [1] finding that health advisors substantially underestimate the level of support for smokefree laws may reflect the tobacco companies’ success in using these strategies (likely supplemented with campaign contributions to and aggressive lobbying of politicians) in Israel.

This is a problem that could be easily solved if the government simply fixed the law, including moving enforcement away from police (never a good choice [30]) to the Ministry of Health and funding an aggressive media campaign to educate the public and promote compliance with the law.

The cost of continuing the weak legislation goes beyond the discomfort of breathing secondhand smoke. Cities, states and nations around the world that implemented comprehensive 100% smokefree laws experienced 10-20% drops in hospital admissions for heart attacks, other cardiac events, stroke, asthma, and other pulmonary events [31], with more comprehensive laws having bigger effects.

As a result, the weaknesses in the current Israeli law are not only protecting multinational tobacco companies’ sales and profits, they are also sending Israeli citizens to the hospital. Right now.

At the same time, following the rest of the world and passing a strong law would keep Israelis healthy.

Right now.

## Authors’ information

Dr. Glantz is a Professor of Medicine, American Legacy Foundation Distinguished Professor of Tobacco Control, and Director of the Center for Tobacco Control Research and Education, where he conducts research on the health effects of secondhand smoke, implementation of effective tobacco control policies, and the tobacco industry as a disease vector.

## Commentary on

Rosen LJ, Rier DA, Connolly G, Oren A, Landau C, Schwartz R: Do health policy advisors know what the public wants? An empirical comparison of how health policy advisors assess public preferences regarding smoke-free air, and what the public actually prefers. Israel Journal of Health Policy Research 2013 2013, 2:20 doi: http://dx.doi.org/10.1186/2045-4015-1182-1120.
